# Facile molten salt synthesis of Li_2_NiTiO_4_ cathode material for Li-ion batteries

**DOI:** 10.1186/1556-276X-9-197

**Published:** 2014-05-01

**Authors:** Yanming Wang, Yajing Wang, Fei Wang

**Affiliations:** 1 School of Chemistry and Materials Science, Huaibei Normal University, Huaibei, Anhui 235000, China

**Keywords:** Lithium nickel titanate, Molten salt method, Cyclability, Li-ion battery

## Abstract

Well-crystallized Li_2_NiTiO_4_ nanoparticles are rapidly synthesized by a molten salt method using a mixture of NaCl and KCl salts. X-ray diffraction pattern and scanning electron microscopic image show that Li_2_NiTiO_4_ has a cubic rock salt structure with an average particle size of *ca.* 50 nm. Conductive carbon-coated Li_2_NiTiO_4_ is obtained by a facile ball milling method. As a novel 4 V positive cathode material for Li-ion batteries, the Li_2_NiTiO_4_/C delivers high discharge capacities of 115 mAh g^-1^ at room temperature and 138 mAh g^-1^ and 50°C, along with a superior cyclability.

## Background

The growing demand for high-energy Li-ion batteries in the development of portable electronic devices and electric vehicles has stimulated great research interest in advanced cathode materials with high voltage and specific capacity. Li_2_MSiO_4_ (M = Fe and Mn) has recently attracted particular attention owing to their high theoretical capacities (>330 mAh g^-1^) and good thermal stability through strong Si-O bond [1-3]. However, the practical discharge capacity is mainly achieved below 3.5 V, resulting in a lower cell energy density. Substituting Si atom for Ti atom leads to another attractive cathode material of Li_2_MTiO_4_ (M = Fe, Mn, Co, Ni) with high theoretical capacity (approximately 290 mAh g^-1^) [4]. The titanate family has a cubic cation disordered rock salt structure, in which the strong Ti-O bond could stabilize the M^3+^/M^2+^ and M^4+^/M^3+^ transition [5,6].

Recently, Küzma et al. [7] synthesized the carbon-coated Li_2_FeTiO_4_ and Li_2_MnTiO_4_ by a citrate-precursor method, which showed the reversible capacity of 123 and 132 mAh g^-1^ at 60°C, respectively. In addition, the reported Li_2_CoTiO_4_/C presented a high discharge capacity of 144 mAh g^-1^ at rate of 10 mA g^-1^[8]. In comparison with Fe, Mn and Co analogues, Li_2_NiTiO_4_ provides much higher discharge voltage plateau near 4.0 V. The electrochemical characterization of Li_2_NiTiO_4_ was initially published in 2004 [9]. In a LiBOB/EC-DMC electrolyte, Li_2_NiTiO_4_ could deliver a charge capacity of 182 mAh g^-1^; however, more than 50% of this capacity was lost after 1 cycle [10]. Kawano et al. [11] reported that Li_2_NiTiO_4_ demonstrated a discharge capacity of 153 mAh g^-1^ at the extremely low rate of 0.32 mA g^-1^ but showed an inferior cycling stability. Li_2_NiTiO_4_ suffers from poor electrode kinetics caused by its intrinsically low ionic and electronic conductivity, leading to a poor electrochemical activity.

In this work, well-dispersed Li_2_NiTiO_4_ nanoparticles are successfully prepared by a molten salt process with a short reaction time. To enhance the surface electronic conductivity and reinforce the structural stability, Li_2_NiTiO_4_ nanoparticles are carbon-coated by ball milling with carbon black. The whole processes are facile and high-yielding, which are promising for industrial application.

## Methods

An equal molar ratio of NaCl and KCl with a melting point of 658°C was used as a molten salt flux. Li_2_CO_3_, Ni (CH_3_COO)_2_ · 4H_2_O, TiO_2_ (5 to 10 nm) and NaCl-KCl (Aladdin, Shanghai, China) in a molar ratio of 1:1:1:4 were well mixed with a mortar and pestle. The mixture was decomposed at 350°C for 2 h, followed by treatment at 670°C for 1.5 h under air. The product was washed with deionized water to remove any remaining salt and dried under vacuum. The as-prepared Li_2_NiTiO_4_ powder was ball-milled with 20 wt.% acetylene black to obtain the Li_2_NiTiO_4_/C composite.

The as-prepared Li_2_NiTiO_4_ was studied by X-ray diffraction (XRD, Rigaku D/Max-2200, Rigaku Corporation, Tokyo, Japan) analysis using Cu Ka radiation (40 kV, 30 mA). The morphologies of the Li_2_NiTiO_4_ and Li_2_NiTiO_4_/C samples were observed by scanning electron microscope (SEM, JEOL JSM-7401 F, Ltd., Akishima, Tokyo, Japan) with an accelerating voltage of 5.0 kV and transmission electron microscope (TEM, JEOL JEM-2100, Ltd., Akishima, Tokyo, Japan) operating at 200 kV. The chemical valence states of transition metals was analyzed by X-ray photoelectron spectroscopy (XPS) acquired with a Kratos Axis Ultra spectrometer (Axis Ultra DLD, Kratos, Japan) using a monochromatic Al Ka source (1,486.6 eV). The amount of carbon was determined from PE 2400II elemental analyzer (Perkin Elmer, USA). The metal content (lithium, nickel, and titanium) of the as-prepared Li_2_NiTiO_4_ was analyzed using an inductively coupled plasma optical emission spectroscopy (ICP-OES) measurements (iCAP6300, Thermo, USA).

Electrochemical tests were performed with CR2016-type coin cells using Li foil as anode. The cathode consisted of 85 wt.% Li_2_NiTiO_4_/C, 5 wt.% Super P carbon black, and 10 wt.% polyvinylidene difluoride binder. An aluminum disk with *Ø* = 1.2 cm was used as current collector in the cathode side, and the pure Li_2_NiTiO_4_ loading is 1.5 mgcm^-2^. The electrolyte was 1 M LiPF_6_ in the mixture of ethylene carbonate (EC) and dimethyl carbonate (DMC) (1:1, *v*/*v*). Galvanostatic charge-discharge measurements were carried out on a LAND CT2001A battery tester (Wuhan, China) in a potential range of 2.4 to 4.9 V at room temperature and 2.4 to 4.8 V at 50°C. The cyclic voltammogram (CV) was measured between 2.4 and 5.1 V using a CHI660D electrochemical workstation (Shanghai, China) with a scan rate of 0.1 mV s^-1^. The specific capacity was calculated based on the mass of pure Li_2_NiTiO_4_ active material.

## Results and discussion

**Figure 1:**
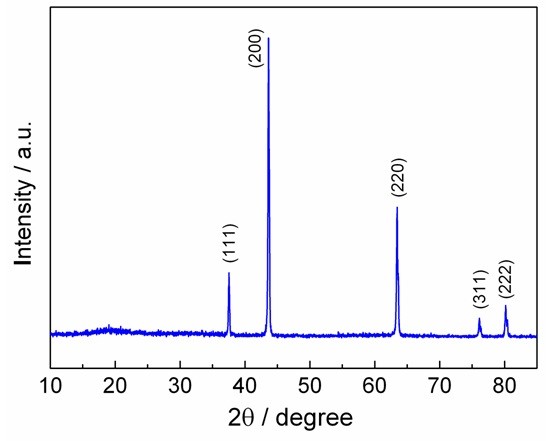
**XRD pattern of Li**_**2**_**NiTiO**_**4**_**.**

Figure 1 shows the indexed XRD pattern of the as-prepared Li_2_NiTiO_4_ powders. Li_2_NiTiO_4_ can be assigned to the rock salt phase with *Fm*-3 *m* space group. The refined cell parameters of *a* = 4.1436(5) Å and *V* = 71.14 Å^3^ are in agreement with previously reported values for Li_2_NiTiO_4_[10,11]. The diffraction peaks are quite sharp, indicating the good crystallinity of the material. The molten salt enables molecular level mixing of reacting species and thus leads to a rapid formation of well-crystallized Li_2_NiTiO_4_ at a moderate temperature. Furthermore, no any residual impunity phases are observed. ICP analysis indicates 2.10:1:0.99 for the atomic ratio of Li/Ni/Ti in the obtained cubic phase, which proves the efficacy of the molten salt method to yield the pure-phase product in a short reaction time.

**Figure 2:**
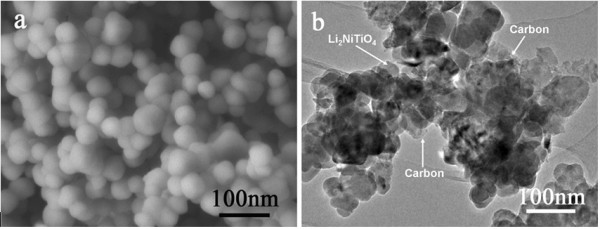
**SEM image of Li**_**2**_**NiTiO**_**4 **_**(a) and TEM image of Li**_**2**_**NiTiO**_**4**_**/C (b).**

The morphology of the as-prepared Li_2_NiTiO_4_ is shown in Figure 2a. The Li_2_NiTiO_4_ powder consists of spherical particles with an average size of ca. 50 nm. Because Ni^2+^ ion may be reduced to metallic Ni by carbon at high temperature, it is difficult to employ polymer pyrolysis method to get carbon coated phase-pure Li_2_NiTiO_4_. In order to improve the poor electronic conductivity, the bare Li_2_NiTiO_4_ nanoparticles are carbon-coated by simple ball milling with conductive carbon. The carbon content in the Li_2_NiTiO_4_/C composite is 19.8 wt.%. The TEM image of Figure 2b demonstrates that the Li_2_NiTiO_4_ nanoparticles are in close contact with the dispersed carbon particles. Thus, the active material particles are interconnected by a carbon network, which is favorable for fast electron transfer and lithium extraction/insertion kinetics.

**Figure 3:**
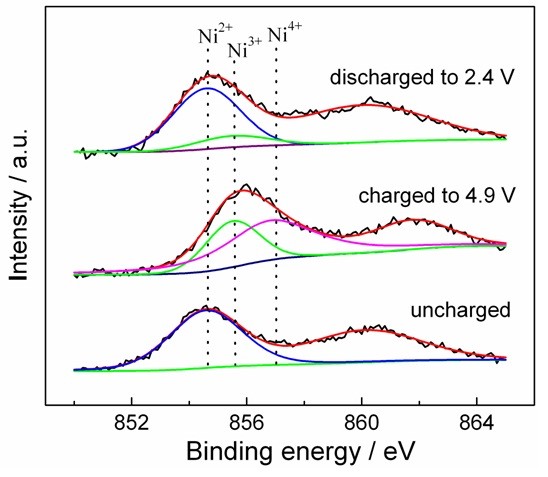
**XPS spectra of Ni 2p**_**3/2 **_**at different charge-discharge state.**

The valence variations of Ni element in the Li_2_NiTiO_4_ electrode during cycling are analyzed by the XPS spectra and fitted in Figure 3. The characteristic binding energy located at 854.6 eV with a satellite peak at 860.5 eV in the Ni 2p_3/2_ XPS spectrum for uncharged Li_2_NiTiO_4_ electrode could be assigned to Ni^2+^ species. The above observations are in agreement with the reported values in LiNi_0.5_Mn_0.5_O_2_, LiNi_1/3_Mn_1/3_Co_1/3_O_2_ and LiNi_0.5_Mn_1.5_O_4_[12-14]. The Ni 2p_3/2_ binding energy gives positive shift when the electrode is charged to 4.9 V, and the two peaks at 855.5 and 856.9 eV are corresponding to the binding energy of Ni^3+^ and Ni^4+^[15], respectively. When discharged to 2.4 V, the Ni 2p_3/2_ binding energy moves back to almost the original position. The best fit for the Ni 2p_3/2_ spectrum consists of a major peak at 854.6 eV and a less prominent one at 855.5 eV. The above results indicate that Ni^2+^ is oxidized to Ni^3+^ and Ni^4+^ during charging, and most of the high valence Ni^3+/4+^ is reduced to Ni^2+^ in the discharge process.

**Figure 4:**
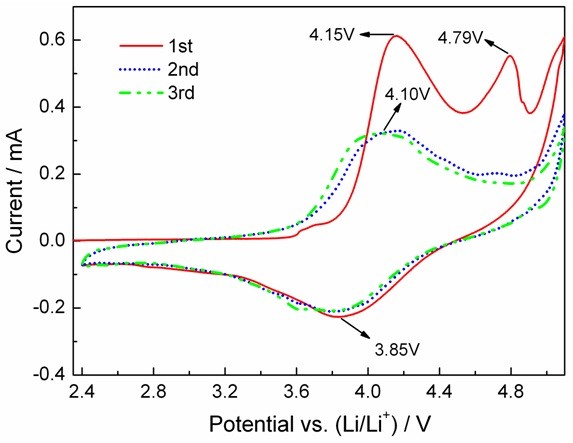
**CV curves of the Li**_**2**_**NiTiO**_**4**_**/C nanocomposite.**

Figure 4 exhibits the CV curves of the Li_2_NiTiO_4_/C nanocomposite. For the first CV curve, a sharp oxidation peak at 4.15 V corresponds to the oxidation of Ni^2+^ to Ni^3+^/Ni^4+^. Another oxidation peak appears around 4.79 V and almost disappears in the second and third cycles, which might be attributed to the electrolyte decomposition and the irreversible structure transitions [8,9]. The wide reduction peak at 3.85 V is assigned to the conversion from Ni^3+^/Ni^4+^ to Ni^2+^. The second and third CV curves are similar, indicating a good electrochemical reversibility of the Li_2_NiTiO_4_/C electrode.

**Figure 5:**
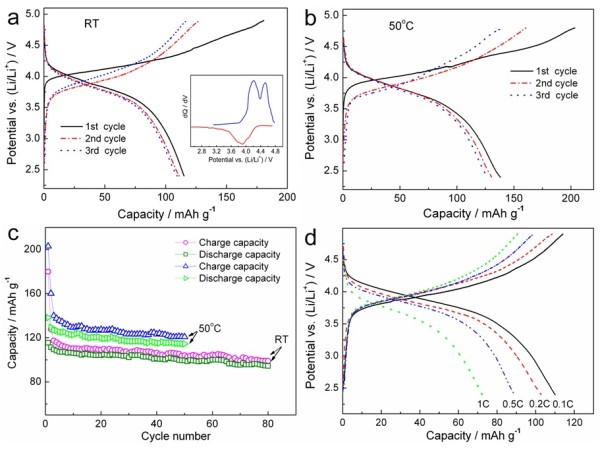
**Electrochemical performances of the Li**_**2**_**NiTiO**_**4**_**/C nanocomposite.** Charge-discharge curves at 0.05 C rate at room temperature **(a)** and 50°C **(b)**, cycling performances at 0.05 C rate **(c)** and rate capability at room temperature **(d)**. The inset in **(a)** shows the dQ/dV plot for the first cycle.

Figure 5a shows the galvanostatic charge-discharge curves of the Li_2_NiTiO_4_/C nanocomposite at 0.05 C rate (14.5 mA g^-1^) under room temperature. The charge/discharge capacities in the first, second, and third cycles are 180/115 mAh g^-1^, 128/111 mAh g^-1^, and 117/109 mAh g^-1^, respectively, with corresponding coulombic efficiencies of 64%, 87%, and 94%. The Li_2_NiTiO_4_/C exhibits superior electrochemical reversibility after the first cycle, which is in accordance with the CV result. The dQ/dV vs. potential plot for the first charge-discharge curve is presented in the inset in Figure 5a. Two oxidation peaks located at 4.2 and 4.5 V in the charge process may be ascribed to the two-step oxidation reactions of Ni^2+^/Ni^3+^ and Ni^3+^/Ni^4+^[10]. However, only one broad peak is observed at approximately 3.9 V belonging to Ni^4+^/Ni^2+^ in the discharge process, which may be resulted from strong hysteresis during the reduction of Ni^4+^ to Ni ^2+^ via Ni^3+^[16].

Figure 5b shows the charge-discharge curves of the Li_2_NiTiO_4_/C nanocomposite at 50°C. It delivers a high initial charge capacity of 203 mAh g^-1^ at 0.05 C rate, corresponding to 1.4 lithium extraction per formula unit. Also, the discharge capacity of 138 mAh g^-1^ is much higher than that tested at room temperature, demonstrating its enhanced electrode kinetics at high temperature. Figure 5c compares the cycling performances of the Li_2_NiTiO_4_/C nanocomposite at room temperature and 50°C. Li_2_NiTiO_4_/C exhibits a stable cycle life after several cycles, and its capacity retentions after 50 cycles are 86% at room temperature and 83% at 50°C. At the end of 80 cycles, Li_2_NiTiO_4_/C retains 82% of its initial capacity with typical coulombic efficiency of 95% at room temperature, displaying a high electrochemical reversibility and structural stability during cycling. Figure 5d presents the rate capability of the Li_2_NiTiO_4_/C nanocomposite at room temperature. The charge rate remains constant at 0.1 C to insure identical initial conditions for each discharge. The Li_2_NiTiO_4_/C retains about 63% of its capacity from 0.05 to 1 C rate. The nanoparticles may reduce Li^+^ diffusion length and improve the ionic conductivity. Moreover, the highly conductive carbon coated on the surface of Li_2_NiTiO_4_ nanoparticles facilitates the rapid electrical conduction and electrode reactions, thus gives rise to capacity delivery and high rate performance.

**Figure 6:**
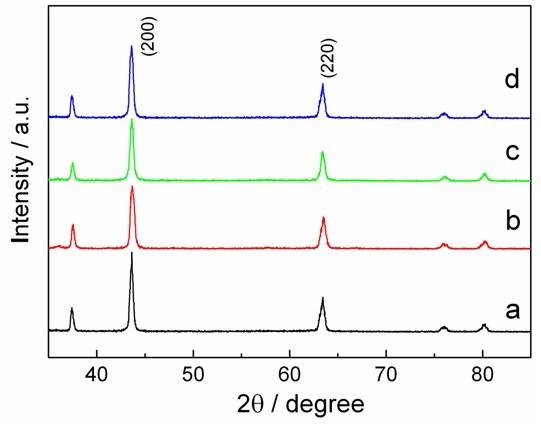
***Ex situ *XRD patterns of the Li**_**2**_**NiTiO**_**4**_**/C electrode.** (curve **a**) Uncharged, (curve **b**) charged to 4.9 V, (curve **c**) discharged to 2.4 V, and (curve **d**) after 2 cycles, at 2.4 V.

In order to investigate the phase change of Li_2_NiTiO_4_ during the charge-discharge process, the *ex situ* XRD of the Li_2_NiTiO_4_/C electrode is employed as shown in Figure 6. XRD peaks corresponding to the Li_2_NiTiO_4_ phase are observed from the pristine cathode sheet. The positions of diffraction peaks are hardly changed during cycling, which indicates that the extraction/insertion of lithium cannot change the framework of Li_2_NiTiO_4_. However, the *I*_220_/*I*_200_ ratio is 0.43 before charging, 0.50 after charging to 4.9 V, 0.48 after discharging to 2.4 V, and 0.47 after 2 cycles. The *I*_220_/*I*_200_ ratios at different charge-discharge states are very close after the first charge, indicating an incompletely reversible structural rearrangement upon initial lithium extraction. Trócoli et al. [10] suggest that lithium ions move from 4a octahedral sites to 8c tetrahedral sites, resulting in an irreversible loss of crystallinity in the material during the first charge process. The above results together with the CV data suggest that the crystal structure can be mainly retained upon the process of lithium extraction/insertion.

## Conclusions

Nanostructured Li_2_NiTiO_4_/C composite has been successfully prepared by a rapid molten salt method followed by ball milling. Cyclic voltammetry together with the *ex situ* XRD analysis indicate that Li_2_NiTiO_4_ exhibits reversible extraction/insertion of lithium and retains the cubic structure during cycling. This Li_2_NiTiO_4_/C nanocomposite exhibits relatively high discharge capacities, superior capacity retentions, and rate performances at room temperature and 50°C. The improved electrochemical performances can be ascribed to the nanoscale particle size, homogeneous carbon coating, and phase retention upon cycling.

## Competing interests

The authors declare that they have no competing interests.

## Authors' contributions

YMW carried out the experiment and prepared the manuscript. YJW gave the advice and guided the experiment. FW conceived the study and revised the manuscript. All authors read and approved the final manuscript.
